# The pattern of growth observed for *Clostridium botulinum* type A1 strain ATCC 19397 is influenced by nutritional status and quorum sensing: a modelling perspective

**DOI:** 10.1093/femspd/ftv084

**Published:** 2015-10-07

**Authors:** Adaoha E. C. Ihekwaba, Ivan Mura, Michael W. Peck, G. C. Barker

**Affiliations:** 1 Gut Health and Food Safety, Institute of Food Research, Norwich Research Park, Colney, Norwich NR4 7UA, UK; 2 Faculty of Engineering, EAN University, Carrera 11 No. 78 – 47, Bogotá, Colombia

**Keywords:** bacterial growth, *C. botulinum*, population dynamics, mathematical modelling, nutrient sensitivity, quorum sensing, toxin regulation

## Abstract

Botulinum neurotoxins (BoNTs) produced by the anaerobic bacterium *Clostridium botulinum* are the most poisonous substances known to mankind. However, toxin regulation and signals triggering synthesis as well as the regulatory network and actors controlling toxin production are unknown. Experiments show that the neurotoxin gene is growth phase dependent for *C. botulinum* type A1 strain ATCC 19397, and toxin production is influenced both by culture conditions and nutritional status of the medium. Building mathematical models to describe the genetic and molecular machinery that drives the synthesis and release of BoNT requires a simultaneous description of the growth of the bacterium in culture. Here, we show four plausible modelling options which could be considered when constructing models describing the pattern of growth observed in a botulinum growth medium. Commonly used bacterial growth models are unsuitable to fit the pattern of growth observed, since they only include monotonic growth behaviour. We find that a model that includes both the nutritional status and the ability of the cells to sense their surroundings in a quorum-sensing manner is most successful at explaining the pattern of growth obtained for *C. botulinum* type A1 strain ATCC 19397.

## Abstract (precis)

*Clostridium botulinum* is one of the significant organisms associated with foodborne hazards, and improved mathematical models which account for complex growth patterns make important contributions to improved food safety.

## Graphical abstract

**Graphical Abstract Figure.**
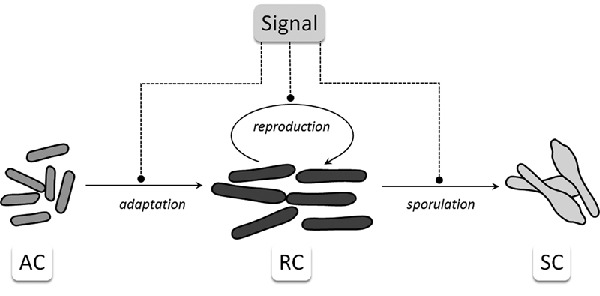
*Clostridium botulinum* is one of the significant organisms associated with foodborne hazards, and improved mathematical models which account for complex growth patterns make important contributions to improved food safety.

## INTRODUCTION

*Clostridium botulinum* is a Gram-positive obligately anaerobic, endospore-forming bacterium that produces a lethal neurotoxin called botulinum neurotoxin (BoNT) (Schantz and Johnson 1992; Peck 2009). BoNTs, highly potent substances with an estimated human lethal dose of ∼30–100 ng (Schantz and Johnson 1992; Peck 2009), are the most powerful toxins known affecting human and animal health. Foodborne botulism when untreated has a high fatality rate, ∼5–10% of cases, and the severity of the disease and the widespread presence and persistence of *C. botulinum* bacteria make botulism a global health concern and a cause for vigilance (Peck 2009). Six phylogenetically distinct clostridia (*C. botulinum* Groups I-IV and some strains of *C. baratii* and *C. butyricum*) produce seven distinct BoNTs (Serotypes A-G) and more than 40 different neurotoxin subtypes (Hill and Smith 2013; Rossetto, Pirazzini and Montecucco 2014; Carter and Peck 2015; Hill *et al*. 2015). These neurotoxins are responsible for human botulism and botulism in a range of other mammals and birds (Peck 2006; Peck, Stringer and Carter 2011).

The structures and the mechanisms of action of BoNTs are reasonably well established (Dolly *et al*. 1984; Melling, Hambleton and Shone 1988; Schiavo *et al*. 1993; Montecucco and Schiavo 1994; Rossetto *et al*. 1994; Marvaud *et al*. 1998; Arndt *et al*. 2006; Adler *et al*. 2012); however, regulation of BoNT production or of the neurotoxin gene (*bont*) expression is not fully understood. Furthermore, the environmental signals that trigger the synthesis of the BoNTs and the regulatory network and actors that control the production of the toxin (and hence expression of the encoding *bont* gene) remain to be elucidated. What is known is that the *bont* gene expression appears to be tightly regulated through positive regulatory elements, including the participation of BotR (Marvaud *et al*. 1998), CLC_1093/CLC_1094, CLC_1914/CLC_1913 and CLC_0661/CLC_0663 two-component signal transduction systems (Connan *et al*. 2012), CodY (Zhang *et al*. 2014) and an Agr quorum-sensing system (Cooksley *et al*. 2010). Negative regulation of *bont* expression by CBO0787/CBO0786 (equivalent to CLC_0842/CLC_0843) has also been reported (Zhang *et al*. 2013). Furthermore, the quantity of BoNT produced is strain dependent and influenced by culture conditions including the nutritional status of the medium (e.g. nitrogen sources), but the precise mechanisms are unknown.

Several *in vitro* methods have been developed and applied to the monitoring of *bont* gene expression in *C. botulinum*, including a gene reporter system, competitive reverse transcription (RT)-PCR and quantitative RT-PCR (Artin *et al*. 2008). One of these studies (Sharma 1999) used an endopeptidase assay developed by Ekong, Feavers and Sesardic (1997) to quantify the effect of incubation temperature and time on neurotoxin formation by *C. botulinum* Group I type A1 strain ATCC 19397. When neurotoxin was first detected within the cells, it was primarily present in the single chain form (initial ratio of single chain to dichain = 100:1). However, as growth proceeded at 25°C, nicking of the single chain led to a steady and substantial increase in the proportion of neurotoxin in the dichain form (final ratio of single chain to dichain = 1:18). At 25°C, as the bacteria entered late exponential phase, and the logarithm of the viable count increased from 6.9 to 8.7 cfu ml^−1^, the concentration of type A toxin in the culture supernatant increased by six orders of magnitude to reach more than 10^5^ MLD_50_ ml^−1^ (see fig. 7 of Peck 2009). A smaller increase in activity was recorded at 37°C. By measuring the neurotoxin concentration within the cells, it was established that the dramatic increase in neurotoxin in the supernatant of strain ATCC19397 was associated with *de novo* synthesis of neurotoxin, and not with the release of neurotoxin loosely attached to the cell or with cell autolysis (Sharma 1999). It is reasonable to conclude that *bont* gene expression is growth phase dependent. Other studies with different strains of *C. botulinum* type A (Call, Cooke and Miller 1995; Bradshaw *et al*. 2004; Couesnon, Raffestin and Popoff 2006; Shin *et al*. 2006; Rao *et al*. 2007) and *C. botulinum* type E (Couesnon, Raffestin and Popoff 2006; Artin *et al*. 2008) have also reported that neurotoxin formation is primarily associated with late exponential and early stationary phase and is, subsequently, drastically decreased during stationary phase. However, these studies examined very few time points during population growth and the *bont* gene expression profile is not fully reported.

The use of mathematical models amenable to simulation and to the analysis of what-if-type scenarios may permit further formulation of hypotheses concerning the gene expression profiles and interactions; additionally, a process of iterative computer simulation could guide future experimentation. Current mathematical models of *C. botulinum* describe beliefs concerning the unknown concentrations of *C. botulinum* spores in the environment (Carlin *et al*. 2004; Peck *et al*. 2010), the uncertain inactivation kinetics for populations of spores at high temperatures and the germination and growth of *C. botulinum* populations for a variety of physico-chemical conditions (Whiting and Call 1993; Graham, Mason and Peck 1996; Fernandez and Peck 1997, 1999; Whiting and Oriente 1997; Zhao, Montville and Schaffner 2002; Peck and Stringer 2005; Malakar, Barker and Peck 2011). These models, which are largely aimed at risk assessment, do not include genetic information beyond group level. These models do not attempt to identify elements of regulatory control which are the key to transferability and an appreciation of cell-to-cell variations (in many situations foodborne botulism may be driven by very few cells so that cell variability is a crucial unknown); opportunities for improved understanding are potentially obscured. Thus, building mathematical models that incorporate some of the genetic and molecular machinery driving the synthesis and release of BoNT requires a description of the growth of the bacterium in a culture, and since *bont* gene expression is growth phase dependent this also builds opportunity for improved translation into hazard assessments.

The objective of this work is to identify a set of plausible options that could be considered when constructing improved models to describe the pattern of growth and toxin production exhibited by *C. botulinum* Group I type A1 strain ATCC 19397. We have used experimental data reported by Sharma (1999) and displayed in fig. 7 of Peck (2009). These sources provide high-quality data, expressed as viable cell count (cfu/ml), for the time course of the population dynamics. Using an analysis of the trends obtained from the time course (showing growth of *C. botulinum* type A1 strain ATCC 19397 at 37°C in viable count), we have generated several working hypotheses on the nature of the ‘signal/s’ that could have generated the pattern of growth observed for type A1 strain ATCC 19397. The growth pattern of type A1 strain ATCC 19397 could be dependent on a signal from either the levels of a nutrient or nutrients in the culture or from a quorum-sensing mechanism. Four mathematical models describing how the signalling species could exert an effect on the adaptation, reproduction and sporulation of strain ATCC 19397 have been generated and tested. The commonly used bacterial growth models, i.e. logistic, Gompertz and Baranyi (Jason 1983; Gibson, Bratchell and Roberts 1987; Bratchell *et al*. 1989; Baranyi and Roberts 1995), are only able to fit monotonic growth curves (Zwietering *et al*. 1990; Buchanan, Whiting and Damert 1997) and were not used in our modelling since they were unsuitable for fitting the pattern of growth observed for strain ATCC 19397. We found that of the four models described, a model that includes both the nutritional status and the ability of the cells to sense their surroundings in a quorum-sensing manner was most able to explain the pattern of growth obtained for strain ATCC 19397.

## MATERIALS AND METHODS

In most situations, *C. botulinum* growth responses are expressed in terms of viable counts or more frequently as optical density measurements (i.e. turbidity measurements which employ a variety of instruments to determine the amount of light scattered by a suspension of cells thus measuring the concentration of cell mass of the population) (Bradshaw *et al*. 2004; Couesnon, Raffestin and Popoff 2006; Shin *et al*. 2006; Rao *et al*. 2007; Artin *et al*. 2008; Cooksley *et al*. 2010; Connan *et al*. 2012). Sometimes optical density measurements are calibrated in terms of traditional microbial enumerations (concentration of colony forming units (cfu)—where the number of cfus is directly related to the viable number of bacteria in the sample) (see Peck 2009, adapted from Sharma 1999) but most often, direct comparison is absent. Since *bont* gene expression is growth phase dependent and the concentration of toxin released in the botulinum growth medium is related to the number of bacterial cells, creating a comprehensive computational model of BoNT production required growth data expressed in terms of microbial number (i.e. reported as viable cell counts (cfu/ml)). For this reason, we chose to use the experimental data reported in Sharma (1999) and in fig. 7 of Peck (2009) because it is expressed as viable cell counts (cfu/ml) and it takes the form of a time course following the population dynamics.

### Culture conditions and growth parameters

The strain considered in the study is *C. botulinum* Group I type A1 strain ATCC 19397 (NCTC 7272). It is a typical *C. botulinum* Group I type A1 strain with the *bont* gene in an ha neurotoxin gene cluster (Carter *et al*. 2009; Carter and Peck 2015). Strain ATCC 19397 was grown in anaerobic (N_2_/CO_2_/H_2_; 85:5:10) peptone-yeast-glucose-starch medium at 37°C (Sharma 1999; Peck 2009). The viable count was determined by plating appropriate dilutions onto VLB agar plates (Peck, Fairbairn and Lund 1992) incubated at 30°C for 48 h under atmosphere of CO_2_/H_2_ (10:90 v/v). The reported data also includes measurements of the amount of toxin released in the supernatant over time. This was quantified using an endopeptidase activity assay developed by Ekong, Feavers and Sesardic (1997). This time series can be used for verifying predictions obtained with models that couple the population dynamics with the toxin production regulation network.

The experimental data can be analysed to identify distinct phases of the culture growth which are then the main input to the formation of hypotheses concerning those physiological aspects of the bacterium that may determine the observed pattern of growth. In this sense, the approach adds to existing empirical models.

Since the objective is to construct models of population growth, and in particular computational models amenable to simulation, we formulated models based on the hypotheses relating to the physiology of the bacteria. We use a reaction-based specification language to describe the evolution of a population of bacterial cells in terms of their growth, reproduction and sporulation. The dependence of the cells on other species, such as nutrient/s and chemical messaging molecules that can be used as signals for quorum sensing, is included as coupled species. The qualitative information is extended by the inclusion of quantitative behaviour, i.e. the kinetics of the reactions, and then transformed into deterministic models represented as systems of coupled ordinary differential equations (ODEs). Simulation is realized via the numerical integration of the ODEs. The whole process is supported by the COPASI modelling and simulation software package (Hoops *et al*. 2006), which takes as its input, reaction-based specifications and provides the simulated time courses for the population dynamics. The kinetics of the reactions are determined empirically, without the usage of any parameter optimization tool, because our purpose is not specifically to find the best fit for the data, but rather to ascertain the appropriate modelling options that qualitatively reproduces the observed behaviours.

Our modelling is based on a compartmentalization of the population of cells in the botulinum growth medium into three distinct groups:

- Adapting cells, denoted by AC, which includes the bacterial cells after their addition to the botulinum growth medium.
- Reproducing cells, denoted by RC, formed by the cells that are actively reproducing.
- Sporulating cells, denoted by SC, which consists of the cells that are committed to sporulation (though not measured in the work of Sharma 1999).

**Figure 1.**
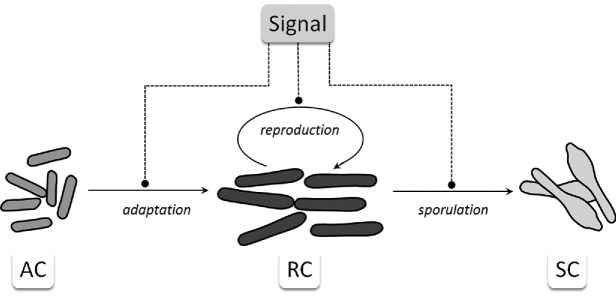
Schematic representation of the population dynamics model. AC is the population of adapting cells, RC the population of reproducing cells and SC of spores. Abundance of the abstract species ‘Signal’ may affect adaptation, reproduction and sporulation. Rounded ending dashed lines are used to represent the influence of the Signal on the population dynamics. This influence can either be a positive or a negative one, depending on the modelling scenario being considered.

In our model, the inoculum of the botulinum growth medium is entirely AC cells, which later evolve to RCs and may commit to sporulation and become SCs. This is illustrated in Fig. 1. With this assumption, we presume that this process is regulated by the ability of cells to sense unknown chemicals in the culture medium, which regulate not just growth, but also toxin production (and gene expression). The population dynamics are coupled to the abundance of an abstract signalling species (which we term ‘Signal’), as shown in Fig. 1. Different hypotheses relating to the nature of the ‘Signal’ have led to the definition of a set of modelling scenarios, which we have presented and analysed in the results section.

## RESULTS

In this section, we present a set of modelling options: four different physiological bases that could account for the observed dynamics of the *C. botulinum* population in the selected strain ATCC 19397 experimental data set. The formal definition of each model is presented, along with the simulation results obtained with the COPASI software tool. We start by analysing the experimental data set that generated the time course of the strain ATCC 19397 population, as reported in fig. 7 of Peck (2009), followed by the modelling options.

### Analysis of the experimental time course

**Figure 2.**
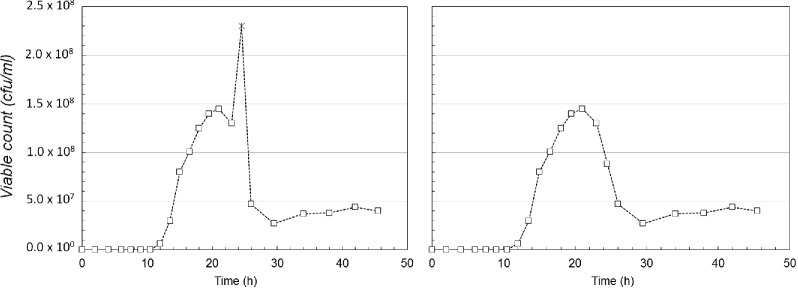
Time course of *C. botulinum* type A1 strain ATCC 19397 population in botulinum assay medium. (Adapted from Sharma 1999 and Peck 2009). Data are the means of duplicate or triplicate determinations taken at 37°C. The empty squares are the experimentally measured data points and the dotted line is the result of the linear interpolation. **(A)** The original data series, and **(B)** the modified data series, used as our reference time series for constructing models.

The growth of strain ATCC 19397 is illustrated in Fig. 2 and is the starting point for our modelling study. The data, reported in Sharma (1999) and in fig. 7 of Peck (2009), are shown on a linear scale in Fig. 2A (data were previously reported on a logarithmic scale). This representation highlights the reported value of the data point at time *t* = 24.5 h (shown with a distinctive marker), which generates a spike in the pattern of the interpolating curve and several inflection points in a small time interval. We have assumed that this point may be erroneous and have replaced it with the average value of the two adjacent experimental data points to give a smoother curve that is illustrated in Fig. 2B.

From a direct observation of the time course in Fig. 2B, we identified the following trends:

- A slow growth phase in the time interval [0,7.5] h, which is often described as the lag phase of the culture.
- An exponential growth phase in the time interval [7.5,13.5] h, where the curve of the logarithm of the counts can be approximated as a straight line.
- A late exponential growth and a stationary phase (time window [13.5,21] h) followed by a rapid decline of the population in the interval [21–30] h.
- Another nearly stationary phase with a possible slow growth tendency during the period [30–45] h.

None of the classical models of bacterial population growth (logistic, Gompertz, Baranyi; Jason 1983; Gibson, Bratchell and Roberts 1987; Bratchell *et al*. 1989; Baranyi and Roberts 1995) can be used to model all the phases observed for the population dynamics (all the classical models produce monotonic increase in the population and a steady state associated with the stationary phase; Zwietering *et al*. 1990). We went further by trying to define possible models of population dynamics, which could explain how differing signalling species could exert an effect on adaptation, reproduction and sporulation. We start by constructing four different mathematical models which could be tested by simulation; four different modelling scenarios, whose qualitative and quantitative analysis could explain the alternating growth phases.

### Modelling option 1—dependence on nutrients

For this modelling scenario, we considered the option that the dynamics of the cell population is affected only by the availability of an essential nutrient or nutrients in the culture medium. Since the abundance of nutrient/s can only reduce over time, in this modelling scenario, we used the basic assumption underlying a logistic model of growth, where a limiting factor shapes the dynamics of a population. In this setting, we did not make any hypotheses as to the specificity of the nutrient/s that regulates the bacterial cell growth. Nonetheless, we did make the assumption that the ‘Signal’ controlling growth is a nutrient/s species in the botulinum growth medium, which is sensed by cells of strain ATCC 19397, and that the concentration of this species correlates positively with the growth of population and negatively with the rate of sporulation.

In this case, the basic population dynamics (as well as in all subsequent modelling scenarios) can be modelled by three reactions:

(1) \begin{equation*} {\rm{AC}}\mathop \to \limits^{{k_1}} {\rm{RC}} \end{equation*}

(2) \begin{equation*}{\rm{RC}}\ \mathop \to \limits^{{k_{2\ \ }}} {\rm{RC}} + {\rm{RC}} \end{equation*}

(3) \begin{equation*}{\rm{RC}}\mathop \to \limits^{{k_{3{\rm{\ \ }}}}} {\rm{SC}} \end{equation*}

which encode, in a formal way, the diagrammatic representation shown in Fig. 1. Furthermore, we include in the model a coupled species called *N*, which represents in an abstract way the nutrient (or nutrients) that determines the limiting effect on growth. Both AC and RC types of cells consume nutrients over time, which we include in the model with two additional reactions:

(4) \begin{equation*}{\rm{AC}} + N\ \mathop \to \limits^{{k_4}} {\rm{AC}} \end{equation*}

(5) \begin{equation*}{\rm{RC}}\ + \ N\mathop \to \limits^{{k_5}} {\rm{RC}} \end{equation*}

The dependence of the growth dynamics that is associated with the consumption of *N* is encoded into the model by a proper selection of the rates of reactions (1), (2) and (3), which were made dependent on the concentration of nutrients, i.e. on *N*. For reaction (1) and (2), the rate grows monotonically with *N* (which is always present in sufficient quantity), and for reaction (3) it decreases monotonically with *N*. Specifically, we used Hill functions to encode into the model the dependence of the rates on the presence or low concentration of *N*. For reaction (1) and (2), the form of the rate is equal to a constant multiplied by a Hill function of the form < *tex* − *mathid* = "*IM*0001" > *N^n^*/(*N^n^* + *K^n^*). This function is used to model rates that decrease in a sigmoidal way as the concentration of nutrients (*N*) diminishes (where the parameter *K* is the inflection point of the sigmoidal curve and the parameter *n* determines the slope of the curve at the inflection point). With this modelling choice, we model both the positive effects of *N* on growth and the inhibitory effect that occurs when the concentration of *N* falls back to a tuneable threshold level.

For reaction (3), sporulation, we used an inverse Hill function, of the form < *tex* − *mathid* = "*IM*0002" > *K^n^*/(*N^n^* + *K^n^*), which also provides a sigmoidal behaviour. The mathematical form of this function allows us to define a sporulation rate that grows as *N* decreases. The rates of reactions (4) and (5) follow conventional mass-action kinetics (i.e. proportional to the product of the concentration of reactants). Values of the rates and of the Hill function parameters used in the model have been provided in the supplemental data file.

We initialized the model at time *t* = 0 with a number of cells in the AC compartment, equal to the size of the inoculum in the experimental setting (Sharma 1999), and we let the initial concentration of species *N* be one (in arbitary units).

**Figure 3.**
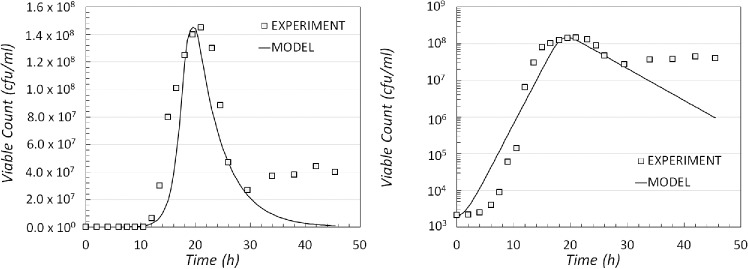
Comparison of experimental data and model output for modelling scenario 1. The result of the simulated dynamics when compared with the experimental data shown on a linear (left) and logarithmic scale (right).

The model was simulated with COPASI and the results of the best fit obtained are shown in Fig. 3, on a linear scale (left) and a logarithmic scale (right). The number of viable cells in the model is defined as the sum of the populations of cells in the AC and RC compartments.

With a hypothesis that considers that dynamics is controlled by a single limiting factor (which decreases over time), the corresponding population dynamics indicates growth and decline (Fig. 3). However, from these results, it is evident that the model does not match the population behaviour in the region later period between time 30 and 48 h, where the number of viable cells appears to achieve some stability (or even growth). Since in this model sporulation is triggered by the lack of essential nutrients and the concentration of nutrients can only decrease over time then sporulation rate can only increase. Thus, it is clear that, in this model, the population size cannot achieve a long-time stationary phase or return to a growth phase.

### Modelling option 2—dependence on nutrients available per cell

In an extension it is natural to consider a scenario that includes the possibility for cells to sense not just the availability of nutrient/s, but also the density of the population in the medium. This is implemented in a model by making the rates of the sporulation reaction (3) dependent on the ratio < *tex* − *mathid* = "*IM*0003" > *N*/RC (i.e. the sporulation reaction is equal to < *tex* − *mathid* = "*IM*0004" > *k* RC *K^n^*/(*K^n^* + (*N*/RC)^*n*^) an inverse Hill function where the regulatory species is < *tex* − *mathid* = "*IM*0005" > *N*/RC). This quantity represents a scaling of the concentration of nutrients with respect to the number of reproducing cells. At the early stage of cell growth, when the reproducing cells’ numbers are low and the concentration of nutrients high, the < *tex* − *mathid* = "*IM*0006" > *N*/RC ratio is large but this reduces quickly as the population grows. The growth of the population also means that the concentration of available nutrient/s diminishes during the exponential phase. As a result of the depletion of nutrient/s, the ratio is eventually expected to fall below a threshold that could in turn trigger sporulation. Due to the drop in nutrient concentration, the population of reproducing cells should decrease quickly, arresting the decreasing trend of the ratio < *tex* − *mathid* = "*IM*0007" > *N*/RC, which in turn decreases the propensity of the cells to sporulate. Ultimately, the culture reaches a state with a small population of surviving cells.

**Figure 4.**
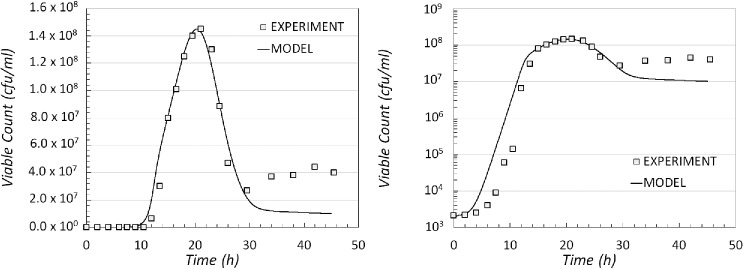
Comparison of experimental data and model output for the modelling scenario 2. The result of the simulated dynamics when compared with the experimental data shown on a linear (left) and logarithmic scale (right).

This model follows from the previous one, with reactions (1)–(5), subject to a change for the sporulation reaction rate (3), to make it dependent on the ratio < *tex* − *mathid* = "*IM*0008" > *N*/RC through an inverse Hill function. The simulated dynamics is compared with the experimental data in Fig. 4 (on linear (left) and logarithmic scales (right)). These figures show a qualitative improvement in agreement between data and model. Nevertheless, this model was still unable to reproduce, quantitatively, the observed dynamics seen between time points 30 and 45 h of the experimental system.

### Modelling option 3—quorum sensing

In order to elucidate the growth pattern observed in the later stages of the culture (between time 30 and 48 h), we consider the possibility that the bacterial cells could be using in a quorum-sensing mechanism, a chemical signal to measure local population density, to regulate sporulation. If this signal is short lived, then it would be possible that, following a drop in the population density, the culture could enter a stage where sporulation is unlikely and a slow growth may resume.

Again a model is constructed using reactions (1)–(3) but in addition a new species, *S*, is introduced to represent the ‘chemical signal’ produced within the cells in culture. The dynamics of the cells following the introduction of *S* is modelled with three reactions:

(6) \begin{equation*}{\rm{RC}}\ \mathop \to \limits^{{k_6}} {\rm{RC}} + S \end{equation*}

(7) \begin{equation*}{\rm{SC}}\mathop \to \limits^{{k_7}} {\rm{\alpha }} \cdot S \end{equation*}

(8) \begin{equation*}S\mathop \to \limits^{{k_8}} \ \emptyset \end{equation*}

Reaction (6) represents, in an abstract way, the production and secretion in the botulinum growth medium, of the signal, *S* whereas reaction (7) models the release of the signal during cell lysis (during sporulation). Parameter < *tex* − *mathid* = "*IM*0009" > α is introduced to allow a proper tuning of the stoichiometry of the reaction, whilst reaction (8) models the degradation of *S*. All the reactions follow a first-order mass action kinetics. Since, in this case, the concentration of the chemical signal *S* is used to affect the kinetics of reactions (2) and (3), the nutrients species *N* (presented in modelling options 1 and 2) are removed.

**Figure 5.**
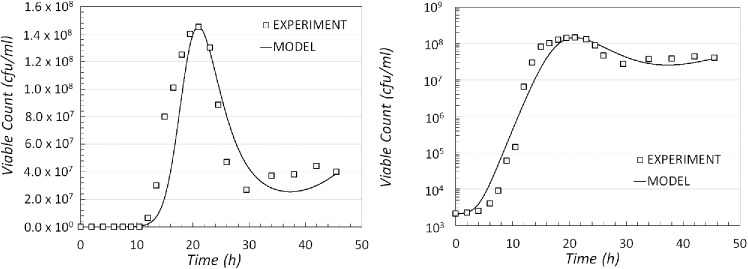
Comparison of experimental data and model output for the modelling scenario 3. The result of the simulated dynamics when compared with the experimental data shown on a linear (left) and logarithmic scale (right).

In this option, the cell reproduction reaction (2) has an inverse Hill type of kinetics, with a rate that decreases as *S* accumulates in the botulinum growth medium, whereas the sporulation reaction (3) has a Hill type kinetics, which increases the rate at which the cells sporulate as the concentration of *S* increases. Using this modelling scenario, there is a better match with the experimental data (Fig. 5).

It is important to note that this model was able to generate dampened oscillations of the population. Indeed, the signal *S* dynamics exhibited a pattern that follows that of the population but is shifted in time, in principle this could drive subsequent phases of reproduction and sporulation.

### Modelling option 4—dependence on nutrients and quorum sensing

It is natural to try to combine the mechanisms that have been introduced to control the population dynamics. In this case, both the nutrients and the quorum-sensing signal influence the population dynamics. Here, the rate at which the cells are reproduced is dependent on the nutrient/s level *N*, whilst the rate of sporulation is dependent on the concentration of the chemical signal, *S*. The scheme representing this complex process includes eight reactions uses:

(1) \begin{equation*} {\rm{AC}}\mathop \to \limits^{{k_1}} {\rm{RC}}\end{equation*}

(2) \begin{equation*}{\rm{RC}}\ \mathop \to \limits^{{k_{2\ \ }}} {\rm{RC}} + {\rm{RC}} \end{equation*}

(3) \begin{equation*}{\rm{RC}}\mathop \to \limits^{{k_{3{\rm{\ \ }}}}} {\rm{SC}} \end{equation*}

(4) \begin{equation*}{\rm{AC}} + N\ \mathop \to \limits^{{k_4}} {\rm{AC}} \end{equation*}

(5) \begin{equation*}{\rm{RC}}\ + \ N\mathop \to \limits^{{k_5}} {\mathop{\rm RC}\nolimits} \end{equation*}

(6) \begin{equation*}{\rm{RC}}\ \mathop \to \limits^{{k_6}} {\rm{RC}} + S \end{equation*}

(7) \begin{equation*}{\rm{SC}}\mathop \to \limits^{{k_7}} {\rm{\alpha }} \cdot S \end{equation*}

(8) \begin{equation*}S\mathop \to \limits^{{k_8}} \ \emptyset \end{equation*}

**Figure 6.**
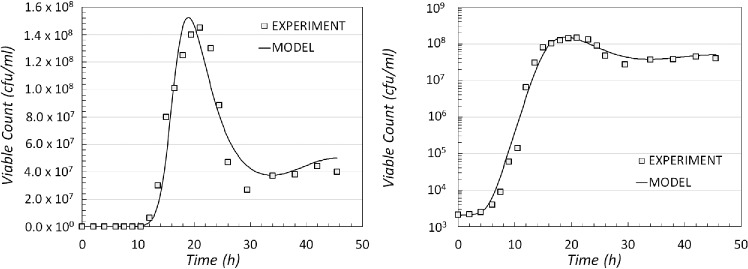
Comparison of experimental data and model output data for the modelling scenario 4. The result of the simulated dynamics when compared with the experimental data shown on a linear (left) and logarithmic scale (right).

Reactions (1) and (2) follow a Hill type kinetics, where *N* is the regulating species, and reaction (3) has Hill type kinetics regulated by species *S*. Using this model it is possible to produce a good fit for the experimental data generated (Fig. 6). Again this model is able to generate oscillatory behaviour in the long-time steady state.

## DISCUSSION

In order to elucidate the genetic and molecular machinery that drive the synthesis and release of BoNT by *C. botulinum*, it was necessary to build a model that described the growth of the bacterium in a culture. Since *bont* gene expression is growth phase dependent and the concentration of toxin released in the botulinum growth medium is related to the number of bacterial cells, creating a comprehensive computational model of BoNT production required an understanding of the population dynamics regulating neurotoxin production and thus modelling of the bacterium's population dynamics.

Four plausible modelling options that could be considered when constructing models describing the growth pattern observed for *C. botulinum* Group I type A1 strain ATCC 19397 in a botulinum growth medium were proposed, implemented and examined. In turn the models captured the dependence of the growth dynamics on (1) the availability of an essential nutrient or nutrients in the culture, (2) the availability of nutrient/s and the density of cells in the growth medium, (3) a chemical signal arising from local populations of cells (quorum sensing), and (4) both the nutrients and the quorum-sensing signal. Traditional models such as logistic, Gompertz and Baranyi (Jason 1983; Gibson, Bratchell and Roberts 1987; Bratchell *et al*. 1989; Baranyi and Roberts 1995), commonly used to model bacterial growth, are unsuitable for describing the growth pattern obtained from *C. botulinum* type A1 strain ATCC 19397 (for reasons explained in Zwietering *et al*. 1990; Buchanan, Whiting and Damert 1997). Based on outputs from simulations of reaction kinetics, we found that the pattern of growth for *C. botulinum* type A1 strain ATCC 19397 could be explained, most fully, by a model that includes both the nutritional status of the medium and the ability of the cells to sense their surroundings in a quorum-sensing manner (i.e. modelling option 4) as drivers of population dynamics.

Although this approach is relatively qualitative, and we have not included detailed methods to compare goodness of fit, it acts as an important precursor to the development of new, quantitative models for toxin production by *C. botulinum*.

## Supplementary Material

Supplementary data are available at FEMSPD online
