# Supplementary material for: The pattern of growth observed for Clostridium botulinum type A1 strain ATCC 19397 is influenced by nutritional status and quorum sensing: a modelling perspective
Source: Pathog Dis. 2015 Oct 7;73(9):ftv084. doi: 10.1093/femspd/ftv084 (PMC4622173; doi:10.1093/femspd/ftv084)
Supplement: Supplementary data are available at FEMSPD online [file femspd_ftv084_index.html]

SUPPLEMENTARY DATA | Pathogens and Disease

## SUPPLEMENTARY DATA

- SUPPLEMENTARY DATA
