# Supplementary material for: The pattern of growth observed for Clostridium botulinum type A1 strain ATCC 19397 is influenced by nutritional status and quorum sensing: a modelling perspective
Source: Pathog Dis. 2015 Oct 7;73(9):ftv084. doi: 10.1093/femspd/ftv084 (PMC4622173; doi:10.1093/femspd/ftv084)
Supplement: Supplementary data are available at FEMSPD online [file Supplemental_DataFile-Final.docx]

## Supplemental Data File

## The pattern of growth observed for *Clostridium botulinum* type A1 strain ATCC 19397 is influenced by nutritional status and quorum sensing: a modelling perspective

Adaoha E. C. Ihekwaba^[[1]](#footnote-1)^ (🖂), Ivan Mura^[[2]](#footnote-2)^, Michael W. Peck^1^, G. C. Barker^1^

## Model Definition Data

Note: all units are a-dimensional

### Modelling option 1 - dependence on nutrients

| Reaction | Mathematical form of the rate | Parameter Values |
| --- | --- | --- |
| $AC \underset{\to}{k_{1}} RC$ | $k_{1}=k\cdot AC\cdot N^{n}/(N^{n}+K^{n})$ | $k=0.4, n=2, K=0.35$ |
| $RC \underset{\to}{k_{2}} RC+RC$ | $k_{2}=k\cdot RC\cdot N^{n}/(N^{n}+K^{n})$ | $k=0.7, n=4, K=0.4$ |
| $RC \underset{\to}{k_{3}} SC$ | $k_{3}=k\cdot RC\cdot K^{n}/(N^{n}+K^{n})$ | $k=0.2, n=6, K=0.1$ |
| $AC+N \underset{\to}{k_{4}} AC$ | $k_{4}=k\cdot AC\cdot N$ | $k=4\cdot{10}^{-9}$ |
| $RC + N \underset{\to}{k_{5}} RC$ | $k_{5}=k\cdot RC\cdot N$ | $k=4.4\cdot{10}^{-9}$ |

### Modelling option 2 - dependence on nutrients available per cell

| Reaction | Mathematical form of the rate | Parameter Values |
| --- | --- | --- |
| $AC \underset{\to}{k_{1}} RC$ | $k_{1}=k\cdot AC\cdot N^{n}/(N^{n}+K^{n})$ | $k=0.025, n=2, K=0.4$ |
| $RC \underset{\to}{k_{2}} RC+RC$ | $k_{2}=k\cdot RC\cdot N^{n}/(N^{n}+K^{n})$ | $k=1.2, n=2, K=0.3$ |
| $RC \underset{\to}{k_{3}} SC$ | $k_{3}=k\cdot RC\cdot K^{n}/((N/{RC)}^{n}+K^{n})$ | $k=0.9, n=4, K=4\cdot{10}^{7}$ |
| $AC+N \underset{\to}{k_{4}} AC$ | $k_{4}=k\cdot AC\cdot N$ | $k=4\cdot{10}^{-10}$ |
| $RC + N \underset{\to}{k_{5}} RC$ | $k_{5}=k\cdot RC\cdot N$ | $k=8\cdot{10}^{-10}$ |

### Modelling option 3 - quorum sensing

| Reaction | Mathematical form of the rate | Parameter Values |
| --- | --- | --- |
| $AC \underset{\to}{k_{1}} RC$ | $k_{1}=k$ | $k=0.025$ |
| $RC \underset{\to}{k_{2}} RC+RC$ | $k_{2}=k\cdot RC\cdot K^{n}/(S^{n}+K^{n})$ | $k=0.9, n=0.35, K={10}^{3}$ |
| $RC \underset{\to}{k_{3}} SC$ | $k_{3}=k\cdot RC\cdot S^{n}/(S^{n}+K^{n})$ | $k=3.5, n=0.35, K=800$ |
| $RC \underset{\to}{k_{6}} RC+S$ | $k_{6}=k$ | $k=2\cdot{10}^{-10}$ |
| $SC \underset{\to}{k_{7}} \alpha\cdot S$ | $k_{7}=k$ | $k={5\cdot10}^{3}, \cdot\alpha=7.5\cdot{10}^{-8}$ |
| $S \underset{\to}{k_{8}} \emptyset$ | $k_{8}=k$ | $k=0.15$ |

### Modelling option 4 - dependence on nutrients and quorum sensing

| Reaction | Mathematical form of the rate | Parameter Values |
| --- | --- | --- |
| $AC \underset{\to}{k_{1}} RC$ | $k_{1}=k$ | $k=0.025$ |
| $RC \underset{\to}{k_{2}} RC+RC$ | $k_{2}=k\cdot RC\cdot N^{n}/(N^{n}+K^{n})$ | $k=2.2, n=0.3, K=0.8$ |
| $RC \underset{\to}{k_{3}} SC$ | $k_{3}=k\cdot RC\cdot S^{n}/(S^{n}+K^{n})$ | $k=2, n=0.475, K={5\cdot10}^{4}$ |
| $AC+N \underset{\to}{k_{4}} AC$ | $k_{4}=k\cdot AC\cdot N$ | $k=4\cdot{10}^{-10}$ |
| $RC + N \underset{\to}{k_{5}} RC$ | $k_{5}=k\cdot RC\cdot N$ | $k=4\cdot{10}^{-10}$ |
| $RC \underset{\to}{k_{6}} RC+S$ | $k_{6}=k$ | $k=8\cdot{10}^{-6}$ |
| $SC \underset{\to}{k_{7}} \alpha\cdot S$ | $k_{7}=k$ | $k={10}^{6}, \cdot\alpha=2.5\cdot{10}^{-4}$ |
| $S \underset{\to}{k_{8}} \emptyset$ | $k_{8}=k$ | $k=0.25$ |

1. (🖂) Corresponding author, -e-mail: [adaoha.ihekwaba@ifr.ac.uk](mailto:adaoha.ihekwaba@ifr.ac.uk)

   Gut Health and Food Safety, Institute of Food Research
   Norwich Research Park, Colney, Norwich, UK
    [↑](#footnote-ref-1)
2. Faculty of Engineering, EAN University
   Carrera 11 No. 78 – 47, Bogotá, Colombia
    [↑](#footnote-ref-2)
